# Cumulus cells conditioned medium facilitates germ cell differentiation from human embryonic stem cells: An experimental study

**DOI:** 10.18502/ijrm.v23i1.18186

**Published:** 2025-03-21

**Authors:** Somayyeh Sadat Tahajjodi, Ehsan Farashahi Yazd, Azam Agharahimi, Fatemeh Akyash, Fatemeh Hajizadeh-Tafti, Jalal Golzadeh, Reza Aflatoonian, Behrouz Aflatoonian

**Affiliations:** ^1^Stem Cell Biology Research Center, Yazd Reproductive Sciences Institute, Shahid Sadoughi University of Medical Sciences, Yazd, Iran.; ^2^Research and Clinical Center for Infertility, Yazd Reproductive Sciences Institute, Shahid Sadoughi University of Medical Sciences, Yazd, Iran.; ^3^Department of Reproductive Biology, School of Medicine, Shahid Sadoughi University of Medical Sciences, Yazd, Iran.; ^4^Department of Endocrinology and Female Infertility, Reproductive Biomedicine Research Centre, Royan Institute for Reproductive Biomedicine, Tehran, Iran.

**Keywords:** Cumulus cells, Conditioned medium, In vitro gametogenesis (IVG), Human embryonic stem cells, Female germ cells.

## Abstract

**Background:**

Studies have shown thatembryonic stem cells can differentiate into germ cells either spontaneously or directed by the defined factors or by applying a co-culture method or conditioned medium of stromal cells.

**Objective:**

Here, Yazd4 (human embryonic stem cells [hESC] line; 46, XX) was used to evaluate the effect of human cumulus cells conditioned medium (CCCM) on *in vitro* germ cell production and development.

**Materials and Methods:**

In this experimental study 2 different complete media were applied, Dulbecco's modified eagle's medium + 20% fetal bovin serum and embryoid body (EB) medium (KnockOut serum replacement- hESC without basal fibroblast growth factor). Thus, there are 2 control groups for spontaneous differentiation (SD) (SD-EB and SD-DM) and 2 types of CCCM (CCCM-DM and CCCM-EB) as test groups. 40% dilution of the conditioned medium was applied according to previous reports.

**Results:**

Reverse transcription-quantitative polymerase chain reaction and immunofluorescent staining assays revealed that between groups with similar complete medium, CCCM provides a better condition for *in vitro* germ cell differentiation from hESCs. Moreover, a comparison between the 2 types of complete media showed that EB medium is more supportive than Dulbecco's modified eagle's medium +20% fetal bovin serum.

**Conclusion:**

CCCM with EB medium as a complete medium provides a better condition for *in vitro* germ cell differentiation from hESCs.

## 1. Introduction

Pluripotent stem cells can differentiate into all types of cells of the body. This makes them suitable for various studies, including embryonic development, tissue engineering, and drug discovery. These cells have embryonic, fetal, and adult origin (1).

Given the enormous potential for embryonic stem cells (ESCs) as pluripotent stem cells, numerous studies have been conducted to differentiate these cells into germ cells. In a study, primordial germ cells (PGCs) derived from mouse embryonic stem cells (mESCs) combined with embryonic gonadal cells and transplanted into testicular tissue to regenerate seminiferous tubules. The results showed that these cells could effectively participate in the process of spermatogenesis (2).

Germ cells could be spontaneously differentiated from human embryonic stem cells (hESCs), as this claim was confirmed by examining the expression of germ cell-specific genes, such as *VASA*, *SCP3*, *GDF9*, and *TEKT1* (3). A study conducted on the differentiation of hESCs into germ cells was successful in identifying the post-meiotic spermatids using sperm-specific antibodies, the expression of oocyte-related genes, and the characterization of follicle-like structures; however, differentiated oocytes with distinctive zona pellucida were not detected (4, 5). Continuous studies in this field have led to a live birth through intracytoplasmic sperm injection of spermatid-like cells, which were differentiated from mESCs, into the ovum (6).

To establish female germ cells, the testicular cell-conditioned medium as a supportive medium was employed to differentiate ESCs into ovarian structures containing oocytes. They demonstrated that the culture of embryoid bodies (EBs) in the testicular cell-conditioned medium could promote the differentiation of EBs into ovarian structures containing potential oocytes (7).

In addition, previous reports show that oogonial-like structures can undergo meiosis, express the SCP3 protein, and induce the adjacent cells to form follicle-like structures (8), an interesting study claimed that, after differentiation of mESCs into PGC-like cells, these precursor cells could be differentiated into oocytes, and the resulting oocytes are capable of producing live birth. The research group co-cultured PGC-like cells with mouse ovarian somatic cells (9).

The studies carried out *in vitro* have shown that cells that are in the neighborhood of female germ cells are involved in their differentiation, such as cumulus cells (10), granulosa cells (11, 12), ovarian somatic cells (13), human fetal gonadal cells (14), and neonatal mouse testis (4). Moreover, the conditioned medium of these cells (10, 15, 16) has been used to confirm their co-culture effect.

In 2 different studies, the impact of the buffalo cumulus cells conditioned medium (CCCM) (10) and human CCCM (hCCCM) (17) on ESCs differentiation was evaluated. The results have shown that the treated conditioned medium of the cumulus cells with ESCs had a positive effect on the differentiation of ESCs into germ cells (10). In addition, recently it was reported that hCCCM supports *in vitro* maturation of germinal vesicle oocytes called *in vitro* gametogenesis compared with the control group which cultured with Dulbecco's modified eagle's medium (DMEM) + 20% fetal bovin serum (FBS) (18).

In this study, Yazd4-hESCs (46, XX) were treated with the hCCCM, and their effect on the differentiation of hESCs into female germ cells was examined by reverse transcription-quantitative polymerase chain reaction (RT-qPCR) and protein markers by immunofluorescence (IF) staining. We selected genes and proteins for investigation that had been shown in previous studies to be expressed in germ cells, particularly in female germ cells (3, 4).

## 2. Materials and Methods

Our study was performed at the Stem Cell Biology Research Center, Yazd Reproductive Sciences Institute, Shahid Sadoughi University of Medical Sciences, Yazd, Iran.

### Yazd4 hESC line

**Figure 1:**
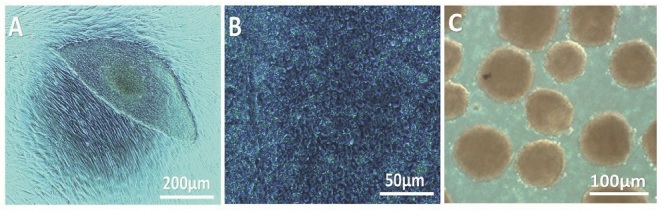
Yazd4 cells and EBs formation. A) Morphology of a Yazd4 colony in a microdrop culture system onto the YhFF#8 feeder layer. B) Morphology of Yazd4 hESCs in higher magnification. C) EBs derived from Yazd4 hESCs. YhFF#8: Yazd human foreskin fibroblast, EBs: Embryoid bodies.

This is an experimental study that used the Yazd4 cell line as a normal female (46, XX) hESC line (Figure 1A, B). This cell line was expanded and characterized using specific markers and genes (19). In sum, the derivation and culture of Yazd4 hESCs were done using a microdrop culture system as explained elsewhere. The difference between Yazd4 derivation procedure and previously reported Yazd hESC lines (20) is the Xeno-free derivation of Yazd4 using Yazd human foreskin fibroblast #8 (YhFF#8) cells (20, 21).

### hCCCM collection 

Cumulus cells were obtained from male factor infertile couples that were entered to assisted reproductive technologies cycle at Research and Clinical Center for Infertility, Yazd Reproductive Sciences Institute, Yazd, Iran. Isolation, culture, and characterization of hCCCM collection were described in our previous study (22).

### Differentiation of hESCs through EB formation

Yazd4 hESCs colonies were cut into smaller pieces by glass pipette and cultured as EBs in 3D nonadherent culture condition. EBs from Yazd4-hESCs were cultured into 4 different conditions: 1) SD-EB: spontaneously differentiation (SD) in 100% EB medium, 2) CCCM-EB: 40% CCCM {40% EB medium (conditioned)} + 60% EB medium, 3) SD-DM: 40% DMEM + 20% FBS (Gibco, US) (not conditioned) + 60% EB medium, and 4) CCCM-DM: 40% CCCM { 40% DMEM + 20% FBS (conditioned) } + 60% EB medium. In each group, EBs were cultured for 4, 7, and 14 days, and half of the medium was replaced every 2 days (Figure 1C).

### RNA isolation, cDNA production, and RT-qPCR

EBs from each group were collected at 12,000 g at 4 C for 10 min by refrigerated microcentrifuge (Sigma, US) at times 4, 7, and 14 after culture. The pellet was suspended in 500 μ l of TRI reagent (Sigma, US) to lyse the cells, completely. The manufacturer's recommended protocol was used for RNA extraction. The quantity of the RNA was determined using a NanoDrop spectrophotometer (Thermo Scientific, UK). DNase treatment kit (Thermo Scientific, UK) was used to remove genomic DNA contamination from RNA samples. First-strand cDNA synthesis was performed using the first-strand cDNA synthesis kit (Takara, Japan). The list of primers used for RT-qPCR is mentioned in table I. RT-qPCR technique was performed using SYBR Premix DimerEraser kit (Takara, Japan) according to the manufacturer's recommended protocol, and the number of PCR cycles was 40. The reactions were performed on applied biosystems (US) instrument in triplicate. Each target gene's threshold value was normalized against the threshold cycle value obtained for the endogenous control B_2_-microglobulin ( β _2_ *M*).

### Immunofluorescent localization technique

IF was applied to evaluate the expression of TRA-2-49 (ab17973, Abcam, US), SSEA1 (SC-21702, Santa-Cruz, US), VASA (ab13840, Abcam, US), GDF9 (ab93892, abcam, US), and ZP3 (SC-398359, Santa-Cruz, US) in Yazd4 hESCs EBs at time 0, 4, 7, and 14 days after culture. IF was conducted as described previously (23). A list of antibodies used for IF staining is mentioned in table II.

**Table 1:** List of primers used for RT-qPCR

**Gene**	**Forward primer (5 ${\;}^{'}$ -3 ${\;}^{'}$ )**	**Reverse primer (5 ${\;}^{'}$ -3 ${\;}^{'}$ )**	**Annealing temp ( C)**	**Product size (bp)**	**Accession number**
*NANOG *	CCCCAGCCTTTACTCTTCCTA	CCAGGTTGAATTGTTCCAGGTC	60	97	(NM_024865.4)
*SOX17 *	TCCGCGGTATATTACTGCAACT	TAGCTCCTCCAGGAAGTGTGTA	60	100	(NM_022454.4)
*DAZL *	ACCACAGTCTTTCGTTTCCAG	TATCCCATTGCTACCGTTCC	58	138	(NM_001351.3)
*DDX4 *	ACAGATGCTCAACAGGATGTTCC	CCCTTTCTGGTATCAACTGATGCA	58	119	(NM_024415.2)
*CTDSPL *	GGCTCCTGATAGGAGGATTTCAT	CCTAAAGTGTTGAGCCACACAA	58	98	(NM_005808.2)
*GDF9 *	CAGAGCTTTGCACTACATGAAGAAG	TGAAGAGCCGAACAGTGTTGT	58	104	(NM_005260.5)
*ZP3 *	CAACAGCATGCAGGTAACTGAC	CGCGGTTAGTCCTCACGAT	60	101	(NM_001110354.2)
*H1T *	AGTGAACAAGGGAATCCTGGTG	TGGTCTTGGCAGAAACTGACTT	60	125	(NC_000006.12)
*PRM1 *	ATAGCACATCCACCAAACTCCT	TTTATTGACAGGCGGCATTGTT	60	129	(NC_000016.10)
* 2M *	AGATGAGTATGCCTGCCGTG	TGCGGCATCTTCAAACCTC	60	106	(NM_004048.2)
RT-qPCR: Reverse transcription-quantitative polymerase chain reaction					

**Table 2:** List of antibodies used for IF staining

**Primary antibody**			**Secondary antibody**		
**Name**	**Dilution rate**	**Catalog number**	**Type**	**Dilution rate**	**Catalog number**
**TRA-2-49**	1/50	ab17973	Anti-mouse IgG (FITC)	1/200	ab6785
**SSEA-1**	1/50	sc-21702	Anti-mouse IgM (TR)	1/200	ab5927
**DDX4 (VASA)**	1/100	ab13840	Anti-rabbit IgG (FITC)	1/200	sc-2359
**GDF9**	1/180	ab93892	Anti-rabbit IgG (FITC)	1/200	sc-2359
**ZP3**	1/50	sc-398359	Anti-mouse IgG (FITC)	1/200	ab6785
IgG: Immunoglobulin G, IgM: Immunoglobulin M, FITC: Fluorescein isothiocyanate, TR: Texas red					

### Ethical Considerations

The participant gave written informed consent. The study was approved by the Ethics Committee of Shahid Sadoughi of Medical Sciences, Yazd, Iran (Code: IR.SSU.MEDICENE.REC.1395.94).

### Statistical Analysis

Normalization of the expressed genes were using human β _2_ *M*, as endogenous control. Relative expression of target genes was calculated as 2 ${\;}^{-\Delta \Delta \text{Ct}\text{mean}}$ . Differences in the normalized expression values between samples were analyzed using a one-way ANOVA statistical test (SPSS version 20). The results are expressed as mean ± SEM, and the statistical significance was set at p < 0.05.

## 3. Results

### Gene expression analysis of hESCs derived germ like cells 

**Figure 2:**
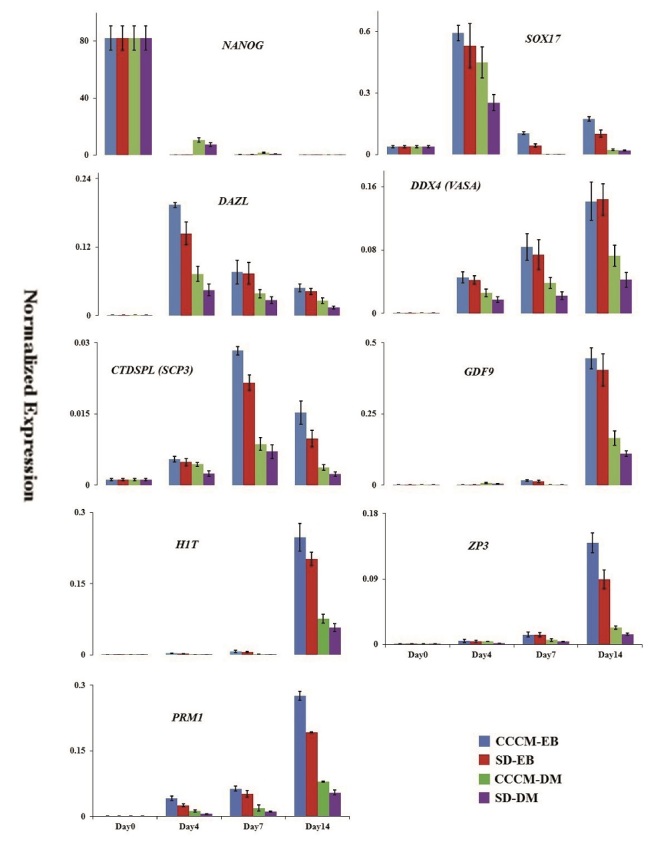
Expression of differentiated EBs analyzed for germ cell development over 14 days of different inducing culture conditions. RT-qPCR was performed for pluripotency (*NANOG*), PGCs (*DAZL, SOX17*), peri-meiotic gonocytes (VASA, SCP3), post-meiotic male germ cells (*H1t, PRM1*), and oogenesis (*ZP3, GDF9*) genes which normalized with internal control gene ( β _2_ *M*). Error bars present statistically significant differences in the target genes (p < 0.05). CCCM: Cumulus cells conditioned medium, SD: Spontaneously differentiation, DM: Dulbecco's modified eagle medium, EBs: Embryoid bodies and RT-qPCR, real time-quantitative reverse transcription polymerase chain reaction.

In the first attempts, SD-EB and CCCM-DM groups were compared, and the results are presented in figure 2 (24). RT-qPCR analysis of EBs collected on days 4, 7, and 14 in these groups showed that the gene expression level in SD-EB was higher than in CCCM-DM. The highest mRNA expression level of *NANOG* was observed in hESCs at day 0. However, low levels of *NANOG* expression were detectable after 4, 7, and 14 days after culture. *SOX17 *and* DAZL *were similar expression patterns highly expressed on day 4. In addition to *SOX17* and* DAZL*, the expression of *DDX4 *(*VASA*)as a *PGCs* gene increased gradually to day 14. The meiotic marker *CTDSPL *(*SCP3*) displayed high expression on day 7. *CTDSPL *(*SCP3*)was expressed on day 14 lower than day 7 and greater than day 4. Female post-meiotic genes including *GDF9* and *ZP3* increased progressively to day 14.

Parallel to the investigation of *in vitro* oogenesis, using gene expression profile assessment, *in vitro* spermatogenesis was also studied using specific genes involved in post-meiotic male germ cell development such as *H1t* and *PRM1*. Like the expression pattern of *GDF9* and *ZP3*, the expression of *H1T* and *PRM1* were higher in SD-EB than in CCCM-DM and increased progressively till day 14. In another investigation which was performed by our group using human testicular cells conditioned medium (5), we observed the same pattern of expression of *H1T* and *PRM1*.

As mentioned before, we had 2 groups (SD-EB and CCCM-DM) with different base mediums. Since the base medium components may affect the results, a resemblance group with the same base medium was created for each group (Figure 2). Comparison of RT-qPCR data between CCCM-EB and SD-EB groups revealed that the expression of *SOX17* on day 7 (p = 0.02), *DAZL* on day 4 (p = 0.04), *CTDSPL* (*SCP3*) on day 7 (p = 0.01), *PRM1* (p = 0.02) and *ZP3* (p = 0.03) on day 14 in CCCM-EB group were significantly more than SD-EB group. Also, between CCCM-DM and SD-DM groups, the expression of *SOX17* on day 4 (p = 0.01), *DDX4 *(*VASA*) (p = 0.03), and *PRM1* (p = 0.04) on day 14 in the CCCM-DM group were significantly more than SD-DM group. Moreover, RT-qPCR data showed that between similar groups with different base medium (EB medium or DMEM + 20% FBS), CCCM-EB and SD-EB groups had significantly more expression than CCCM-DM and SD-DM (p < 0.001). Interestingly, the expression of male germ cell-specific genes was higher in the CCCM-EB group than in the other groups.

### *In vitro* gametogenesis determination using specific antibody markers

**Figure 3:**
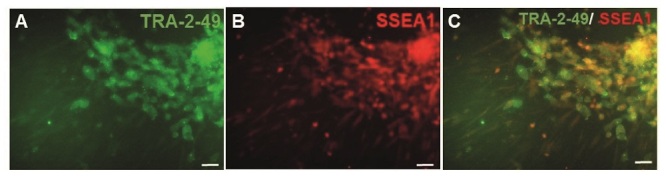
Co-localization of TRA-2-49 and SSEA1 in some parts of early differentiated Yazd4 hESCs colonies using IF staining. A) TRA-2-49, B) SSEA1, and C) Merged. Scale bars: 50 μ m. IF: Immunofluorescent, hESCs: Human embryonic stem cells.

**Figure 4:**
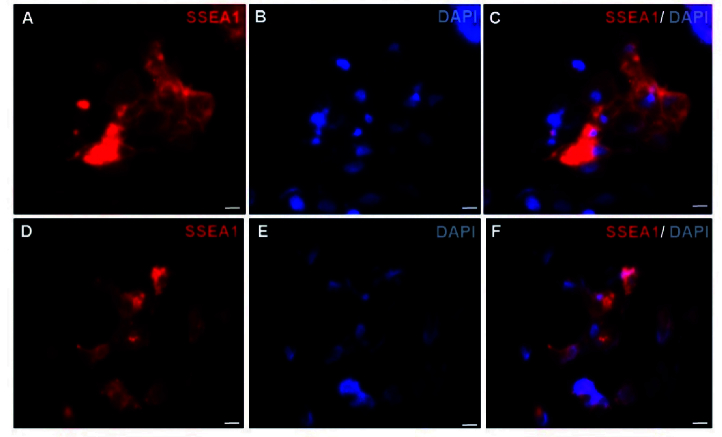
Characterization of PGC-like cells and gonocyte-like cells using IF staining. A-C) Expression of SSEA-1 in CCCM-EB group in day 4 EBs. A) SSEA1, B) DAPI, and C) Merged. Scale bars: 50 μ m. D-F) Expression of SSEA1 in SD-EB group in day 4 EBs. D) SSEA1, E) DAPI, and F) Merged. Scale bars: 50 μ m. IF: Immunofluorescent, EBs: Embryoid bodies, CCCM: Cumulus cells conditioned medium, SD: Spontaneously differentiation.

**Figure 5:**
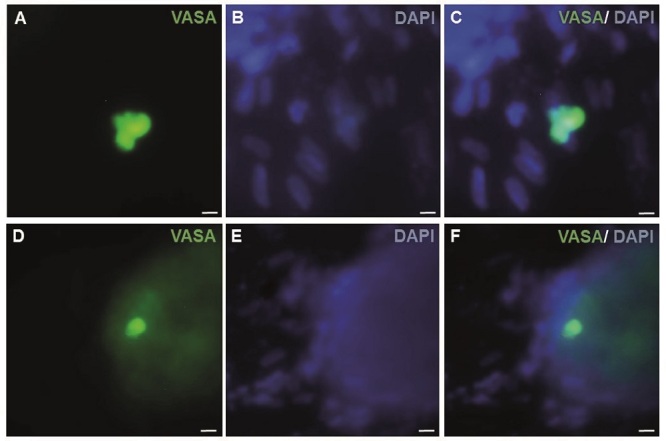
Characterization of pre-meiotic cells using IF staining. Expression of VASA in the differentiated EBs within 7 days after differentiation. A) VASA, B) DAPI, and C) Merged in the CCCM-EB group. Scale bars: 25 μ m. D) VASA, E) DAPI, and F) Merged in the SD-EB group. Scale bars: 50 μ m. IF: Immunofluorescent, EBs: Embryoid bodies, CCCM: Cumulus cells conditioned medium, SD: Spontaneously differentiation, VASA; DDX4, DEAD box polypeptide 4.

As SSEA1 is not expressed in hESCs, but expressed in both PGCs and human embryonic germ cells (hEGCs) co-expression of SSEA1 and TRA-2-49 indicates the formation of PGCs and hEGCs. Co-localization of TRA-2-49 and SSEA1 in some parts of early differentiated cells were observed indicating PGC formation (Figure 3). Also, this co-expression may refer to the presence of hEGCs; however, these cells were not expanded further. The results of IF staining in comparison between CCCM and SD groups showed that expression of SSEA1 (Figure 4) and VASA (Figure 5), protein markers in germ cells, were higher in CCCM groups than in SD groups. However, GDF9 and ZP3 were observed in none. Similar to our study, in a previous study some late markers of oogenesis were detected at the genomics level but not at the proteomics level (4).

### Morphological study of differentiated EBs

**Figure 6:**
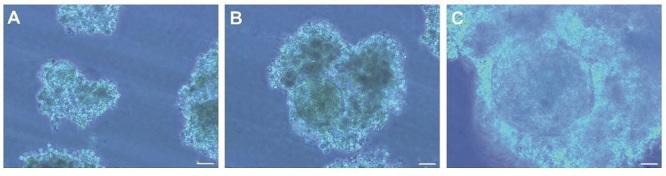
Oocyte-like structures in embryoid bodies (EBs) fromYazd4 hESCs. However, without doing any analysis/expression using a specific signature it would not be a correct statement, and there could be some structural changes during the culture. Scale bars: A) 200 µm, B) 100 µm, C) 50 µm. EBs: Embryoid bodies.

We observed some follicle-like structures (size: around 20–25 µm) in Yazd4 hESCs EBs (Figure 6). These structures contained a round shape large cell (size: around 20–25 µm) in the center that was surrounded by a mass of cells, similar to those which were called in a previous report as follicle-like structures (8).

## 4. Discussion

In this study, the impact of culture with hCCCM on the differentiation of hESCs into female germ cells was compared between SD and CCCM groups by measuring the relative expression levels of *NANOG*, *SOX17*, *DAZL*, *DDX4* (*VASA*), *CTDSPL* (*SCP3*), *GDF9*, *ZP3 *by RT-qPCR on days 0, 4, 7, and 14, cultured with the same base media (either EB or DMEM + 20% FBS). The expressions of TRA-2-49, SSEA1, VASA, GDF9, and ZP3 protein markers were analyzed using the IF staining. RT-qPCR results for the relative expression of the pluripotency gene (*NANOG*), as well as specific genes for pre-meiotic and female germ cells, showed a common expression pattern in all 4 groups, similar to previous studies (3, 4, 25).

The highest expression of the *NANOG* gene was observed in Yazd4 colonies on day 0. The expression of this ESCs-specific gene indicates the undifferentiated state and maintenance of their pluripotency (26, 27). A previous study has shown that the expression of the *SOX17* gene is considered a primary marker at the onset of PGCs differentiation in humans. In the present study also, the expression of *SOX17* in EBs on day 4 was more pronounced than on days 7 and 14, which may indicate the initiation of differentiation of Yazd4 hESCs-EBs toward germ cells (25). However, in another study by our group using Yazd2 hESCs the highest expression of *SOX17* was detected after 7 days of differentiation (5). This shows that each cell line, especially Yazd2 and Yazd4 with male and female chromosomal contents may show its differentiation pattern (28, 29).

The function of the *DAZL* gene is mainly involved in the formation of PGCs, but its expression has been shown to play a significant role in the late stages of meiosis and the development of haploid gametes in collaboration with *DAZ* and *BOULE* genes (30). In this study, similar to previous studies, *DAZL* gene expression on day 4 was more significant than on days 7 and 14, which is consistent with the defined role of this gene in the formation of PGCs (3, 4). Previous studies have shown that the expression of the *DDX4* (*VASA*), as a highly specific gene in germ cells, is observed in PGCs migrating to the gonads and subsequently in primary germ cells formed in the fetal ovary and testis, and it is more expressed in spermatozoa and mature oocytes (31, 32). In this study, the expression pattern of *DDX4* (*VASA*) on days 4, 7, and 14 was incremental, which is consistent with the results reported by Clark et al. and also with the *in vivo* expression levels of this gene (3).

The *CTDSPL* (*SCP3*) gene expresses a synaptonemal protein complex that by creating synapses between homologous chromosomes at the prophase I stage of meiotic division, results in crossing-over and consequently recombination (33). In our previous study, we observed the highest expression of this gene on day 7. However, the expression of this gene on day 14 was also significant and greater than that on day 4. Given the incremental expression of specific pre-meiotic genes, such as *SOX17* and *DAZL* on day 4, it seems that the increased expression of this gene after day 4 was normal. These findings were in line with the results reported by Aflatoonian et al. which showed the incremental expression of this gene up to day 14 (4, 5).

In another study, differentiation of hESCs into germ cells was supported by various factors, including bone morphogenetic protein 4, retinoic acid, and conditioned medium derived from the neonatal mouse testis. The expression of germ cell-specific genes, including *DAZL*, *DDX4* (*VASA*), and *CTDSPL* (*SCP3*), as well as the expression of female germ cell-specific genes, including *GDF9* and *ZP1 *and male germ cell-specific genes, including *PRM1*, a post-meiotic male germ cell genes which are involved in sperm head condensation, and *H1T* were assessed. The results of the expression of these genes in the SD group are consistent with the results of the present study (4, 5).

In our study, hCCCM with 2 different base media was used to investigate its impact on the differentiation of hESCs into female germ cells. Comparison of RT-q-PCR results between CCCM-EB and SD-EB groups showed that relative expression of *SOX17* on day 7, *DAZL *on day 4, *CTDSPL* (*SCP3*) on day 7, *PRM1* and *ZP3* on day 14 was significantly higher in the CCCM-EBs group than that of the SD-EB group. Also, a comparison of RT-q-PCR results between CCCM-DM and SD-DM groups showed that the relative expression of *SOX17* on day 4, *PRM1,* and *DDX4* (VASA) on day 14 was significantly higher in the CCCM-DM group than the SD-DM group, which indicates the impact of the base medium and serum and their role within the differentiation medium composition (34).

Similar to the present study, other studies used cumulus cells, granulosa cells, and the conditioned medium of these cells and reported comparable results with the current research. In 2 other studies, the impact of co-culture of granulosa cells, mouse ovarian stromal cells, and the conditioned medium of each of the 2 cell lines on the differentiation of mESCs and hESCs into female germ cells were investigated and the results showed that the expression of germ cell markers including specific markers of female germ cells was significantly more than control group (11, 12).

In another study, the effect of hCCCM on the differentiation of Wharton's jelly derived mesenchymal stem cells into oocyte-like cells was examined. The results showed that the hCCCM could cause the expression of germ cell genes such as *VASA*, *SCYP3*, *ZP1*, *ZP3,* and *GDF9*, and was found to be increased than the control (17).

In another study conducted by Shah and colleagues, the effect of the conditioned medium of buffalo cumulus cells on the differentiation of buffalo ESCs into germ cells was determined. The method of culturing cumulus cells and CCCM collection was the same in their report and the current study. Shah and colleagues compared the concentrations of 10%, 20%, and 40% conditioned medium of buffalo cumulus cells to determine the best concentration used for CCCM. For this purpose, EBs were cultured with each of these concentrations for 14 days, and they examined the expressions of *NANOG*, *DAZL*, *VASA*, *TNP2*, *PRM2*, *GDF9*, *ZP2,* and *ZP3* on days 4, 8, and 14. The results showed that the optimum concentration for CCCM is 40%, and this concentration was applied for CCCM in our study. Results of RT-qPCR for expression of germ cell genes such as *DAZL*, *VASA*, *TNP2*, *PRM2*, *TEKT1*, *GDF9*, *ZP2*, and *ZP3* showed that expression of these genes was significantly higher in the group cultured with CCCM than the spontaneous differentiation group. Consistent with the current study, the results obtained from IF for VASA markers were similar to the study of Shah and co-workers (10).

## 5. Conclusion

This study showed that neighboring cells (here cumulus cells) have an impact on supporting differentiation of hESCs into targeted committed cells (here germ cells were targeted) by secreting specific factors inside this medium which could improve the outcomes. Also, a comparison of 2 different base media (DMEM + 20% FBS and EBs) showed that the EBs medium is a more suitable medium for differentiating hESCs into female germ cells.

##  Data Availability

In this study, the data are available from the corresponding author on reasonable request.

##  Author Contributions

Drafting of the manuscript, culture of human cumulus cells, collection of CCCM, culture, expansion, differentiation of Yazd4 hESC line into germ cells, performing IF and qPCR techniques: SS. Tahajjodi. qRT-PCR and molecular biology management: R. Aflatoonian and E. Farashahi Yazd. Preparation of cumulus cells: A. Agharahimi. Contribution in culture and expansion of Yazd4 hESCs line: F. Hajizadeh-Tafti, J. Golzadeh, and F. Akyash. Study design, supervision, writing the final version of the manuscript for submission: B. Aflatoonian.

##  Conflict of Interest

The authors declare that they have no conflict of interest.
